# ArboItaly: Leveraging open data for enhanced arbovirus surveillance in Italy

**DOI:** 10.3389/fphar.2024.1459408

**Published:** 2024-09-23

**Authors:** Francesco Branda, Marta Giovanetti, Giancarlo Ceccarelli, Massimo Ciccozzi, Fabio Scarpa

**Affiliations:** ^1^ Unit of Medical Statistics and Molecular Epidemiology, Università Campus Bio-Medico di Roma, Rome, Italy; ^2^ Department of Sciences and Technologies for Sustainable Development and One Health, Università Campus Bio-Medico di Roma, Rome, Italy; ^3^ Instituto Rene Rachou, Fundação Oswaldo Cruz, Belo Horizonte, Brazil; ^4^ Climate Amplified Diseases and Epidemics (CLIMADE), Brasilia, Brazil; ^5^ Department of Public Health and Infectious Diseases, University Hospital Policlinico Umberto I, Sapienza University of Rome, Rome, Italy; ^6^ Department of Biomedical Sciences, University of Sassari, Sassari, Italy

**Keywords:** arboviruses, Italy, open data, west nile, chikungunya, dengue, public health, surveillance

## 1 Introduction

Arboviruses, or arthropod-borne viruses, constitute a significant group of pathogens that frequently induce diseases in diverse global regions (Troppens, 2024). These viruses are predominantly transmitted through biological interactions between infected hematophagous arthropods and susceptible vertebrate hosts. Notably, the rapid and concerning reemergence of these viruses in the 21st century is attributed to several factors, including viral adaptation to new vectors, global population growth, urbanization of forest areas, climate change, globalization, and increased population mobility (Troppens, 2024; Weaver, 2013). The mosquito species *Aedes aegypti* and *Aedes albopictus* (Kraemer et al., 2019; Nakase et al., 2023) are among the most prominent vectors of arboviruses, transmitting major pathogens such as Zika (Giovanetti et al., 2020), dengue and chikungunya (Mohapatra et al., 2024a), yellow fever (YFV) (Giovanetti et al., 2023). These viruses impose significant health and economic burdens globally. The widespread distribution of *Aedes spp*. Mosquitoes now extends across all continents, including North America and Europe, a geographical expansion facilitated by globalization and anthropogenic environmental changes, particularly those associated with climate change. Furthermore, *Culex spp*. Mosquitoes serve as vectors for medically significant arboviruses to animals, including humans, such as West Nile Virus (WNV) (Mingione et al., 2023), Japanese encephalitis virus (Unni et al., 2011), St. Louis encephalitis virus (Kopp et al., 2013), Usutu virus (Roesch et al., 2019), and Murray Valley encephalitis virus (Knox et al., 2012). These and other viruses of the Japanese encephalitis serocomplex are typically spread via infected migratory birds that act as amplifying hosts, influencing the geographical dispersion patterns of these arboviruses. The term “arbovirus” is not a taxonomic classification but encompasses viruses with diverse characteristics from various viral families. Over 500 species of arboviruses are known, and this number continues to grow with advances in DNA sequencing technologies facilitating the genetic characterization of viruses. It is estimated that only about 1% of the world’s total arbovirus diversity is currently identified.

The emergence of infectious diseases poses significant public health challenges globally, with recent outbreaks highlighting the urgent need for effective surveillance and response strategies. In India, the recent surge of scrub typhus (Mohapatra et al., 2024b), particularly in the Sundergarh district of Odisha, has raised alarms as it coincides with outbreaks of other infectious diseases such as leptospirosis. Scrub typhus, a rickettsial infection transmitted by chiggers, manifests with symptoms that can lead to severe complications if not diagnosed and treated promptly. The increasing incidence of scrub typhus, alongside other vector-borne diseases, underscores the impact of environmental changes, urbanization, and population mobility on disease transmission dynamics.

With the rapid global dissemination of these viruses, there is a critical need for robust public data and genomic surveillance systems. These systems are vital for monitoring these diseases and facilitating effective public health responses to prevent large-scale epidemics. The emergence and dispersion of arboviruses are thus significant public health concerns, necessitating comprehensive strategies for surveillance and control. Climate change is leading to an increase in extreme weather events, such as severe droughts and floods, across various continents. With ongoing global warming, these pathogens are progressively moving northward. Since 2007, several European countries, including France, Spain, and Croatia, have faced arboviral epidemics (Zatta et al., 2023; Herrero-Martínez et al., 2023; Pem-Novosel et al., 2015). This underscores the urgent need for the European Union to proactively address the evolving threat posed by these diseases as they extend further into traditionally non-endemic regions.

Such tropical diseases are expected to become more prevalent in Europe and North America soon. Therefore, it is crucial to enhance our understanding of the genetic variants of pathogens with epidemic and pandemic potential to ensure their rapid identification and containment. Factors such as increased connectivity between endemic regions, urbanization, population growth, and climate change are likely to amplify the potential for sustained outbreaks, yet substantial evidence is still required.

In recent years, Italy has faced an increasing number of arboviral disease outbreaks, especially concerning WNV. Despite this growing threat, the fragmented nature of data, often scattered across different sources and formats, has limited the ability to conduct timely analyses, hampering public health efforts to respond quickly to outbreaks. To fill these critical gaps, this paper presents ArboItaly, the first comprehensive and centralized repository dedicated to arboviral disease surveillance in Italy, which not only consolidates epidemiological data from different official sources, but also standardizes and integrates information on different hosts (humans, mosquitoes, equids, and birds) as in the case of West Nile, facilitating the identification of spatiotemporal trends in the spread of a given arbovirus and enabling public health authorities to target control measures more efficiently and allocate resources to high-risk areas.

A paradigm shift in the approach to the growing threat of arboviral diseases is needed, emphasizing the need for greater investment in public health infrastructure. Enhanced surveillance systems, such as ArboItaly, should be supplemented with genomic data to better understand the evolution of arboviruses and their adaptation to new environmental conditions. As arboviral diseases continue to expand into previously unaffected regions, international cooperation and information sharing will be key to mitigating their global impact. Strengthening global surveillance networks will ensure that countries such as Italy, which face similar challenges, can learn from each other’s experiences and best practices, promoting a collaborative approach to disease prevention and control. Integrating environmental monitoring with health data analysis is particularly crucial in the context of climate change, which is changing the geographic range of mosquito vectors and increasing the likelihood of arboviral outbreaks in temperate regions. By using predictive models that incorporate climate data along with epidemiological and genomic information, public health systems can anticipate epidemics and implement preventive measures before they occur. This proactive approach is essential for managing the evolving threat posed by arboviruses, as traditional strategies, such as vector control through insecticides and education and awareness campaigns, are often insufficient in the face of rapid ecological changes and the spread of disease. Through the active and strategic promotion of international collaboration, we can build more resilient and adaptable public health systems that can promptly and competently address emerging challenges and growing risks related to arboviruses, ensuring a coordinated and effective global response.

## 2 Methods

**FIGURE 1:**
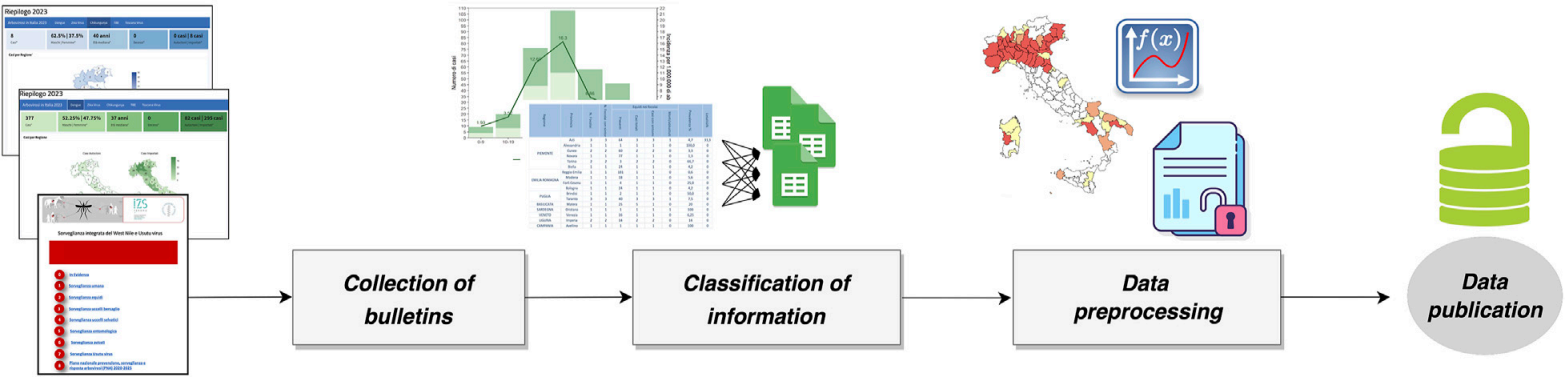
Schematic overview of the key steps to build the dataset.

Our goal was to develop a centralized dataset that stores key information about arboviruses in Italy. Figure 1 summarized the data production process covering from the digitalization to the release of ArboItaly.

The first step (i.e., *Collection of bulletins*) was to systematically collect the publications from the Italian National Institute of Health (ISS) available on the EpiCentro webpage (https://www.epicentro.iss.it/arbovirosi/bollettini). To do so, we reviewed a total of 192 reports (https://www.epicentro.iss.it/westnile/bollettino) published in PDF format regarding WNV from 27 September 2012, to 4 September 2024. For dengue and chikungunya, we utilized data shared through a dashboard from 2015 to 6 August 2024 (https://www.epicentro.iss.it/arbovirosi/dashboard).

Once the bulletins were collected, the next step (i.e., *Classification of information*) was to classify the information according to the type of arbovirus being treated. This process involved extracting and categorizing key information, such as demographic information about the age and gender of cases, geographic information at the highest resolution available down to the province level, and other relevant epidemiological information (e.g., type of infection, species affected). We adopted a mixed system combining automatic scripts and manual extraction to transform the data into a structured format that could be easily used for analysis. Automatic scripts, including Tabula (https://tabula.technology/) and the online services such as WebPlotDigitizer (https://automeris.io/) and Wayback Machine (https://wayback-api.archive.org/) were used to extract tabular information from PDFs of WNV bulletins, digitize data from dashboard charts, and reconstruct the history of shared dengue-related data, respectively. However, bulletins also contain sections of unstructured text that cannot be easily extracted with automated tools. Therefore, we supplemented our automated process with manual extraction of these text sections to ensure that important information is not overlooked, and that all data are collected accurately.

The third and final step in the process before releasing the dataset publicly was the pre-processing of the digitalized data. We addressed various challenges related to data quality and consistency, implementing rigorous quality controls to ensure the accuracy of the final dataset. This included cleaning the data to remove any errors or inconsistencies. For example, due to reporting delays and possible measurement errors, weekly WNV cases may have negative values, and this is not correct because cumulative counts must meet a monotonicity constraint. As a result, we chose to set as “Not Available” (“NA”) all negative values of weekly cases (“new_cases”) whenever the cumulative number of cases for the current week (“total_cases”) was lower than that of the previous week. A standardized procedure was also adopted to code geographic information following ISTAT nomenclature, which involved several steps: (i) coding all geographic data using official ISTAT codes for regions and provinces to ensure exact correspondence with official administrative divisions; (ii) adding geographic coordinates (i.e., longitude and latitude) for each unit to facilitate spatial analysis and mapping; (iii) managing geographic ambiguities by using unique identifiers for localities with the same name; and (iv) implementing a consistent schema with standardized prefixes and suffixes to improve readability and integration with other datasets.

## 3 Data description

To accurately track epidemiological trends of WNV infections in Italy at regional and provincial levels, we organized the collected data according to the different hosts monitored, i.e., humans, mosquitoes, equids, wild and targets birds. In the case of humans, we acquired and analyzed detailed data, including the age of patients and the type of infection (e.g., neuroinvasive, fever, blood donor), allowing a thorough understanding of the spread and impact of the virus in local communities. Mosquitoes, the main vectors of WNV, were monitored through regular collection and testing to identify new cases and outbreaks. In addition, monitoring of equids and birds (both target and wild), has been crucial, focusing on specific species and geographic areas most at risk to identify outbreaks and understand the dynamics of WNV transmission in wildlife. Monitoring dengue and chikungunya infections, on the other hand, involves weekly surveillance of reported cases, with a focus on new cases and related deaths to identify trends in incidence and spread over time. In addition, we subdivided the data according to several key parameters, such as gender, age, and type of infection (autochthonous or imported), to obtain a comprehensive view of the demographic profile of infections. Finally, the subdivision of region cases allows for precise mapping of the most affected areas to develop effective policies in the highest risk areas.

Table 1 provides a concise overview of the structure and main contents of the dataset. The README file of the GitHub repository includes detailed code snippets that describe how to import and manipulate the data. Brief instructions to assist other researchers with reuse of the data are available in Supplementary Material.

**TABLE 1:** Overview of the dataset structure and main contents.

Subject	Public health and health policy
Specific subject area	Infectious diseases and virology
Data accessibility	Public repository: GitHub ( https://github.com/ ) Repository name: arbovirus Direct URL to data: https://github.com/fbranda/arbovirus License: CC-BY-4.0
Files and fields	1) wn-ita-regions-human-surveillance-yyyy.csv • url_bolletins: URL to the surveillance bulletins • data: Date of the reported data • code_region: Code identifying the region (ISTAT nomenclature) • name_region: Name of the region • lat: Latitude coordinates of the region • long: Longitude coordinates of the region • new_cases: Number of new weekly cases reported • total_cases: Total number of cases reported since the beginning of surveillance • type_infection: Type of infection reported 2) wn-ita-provinces-human-surveillance-yyyy.csv • url_bolletins: URL to the surveillance bulletins • data: Date of the reported data • code_region: 2-digit code identifying the region (ISTAT nomenclature) • name_region: Name of the region • code_province: 3-digit code identifying the province (ISTAT nomenclature) • name_province: Name of the province • abbreviation_province: Province 2-letter code (ISTAT nomenclature) • lat: Latitude coordinates of the province • long: Longitude coordinates of the province • age: Age of reported cases • new_cases: Number of new weekly cases reported • total_cases: Total number of cases reported since the beginning of surveillance • type_infection: Type of infection reported 3) wn-ita-mosquito-surveillance-yyyy.csv • url_bolletins: URL to the surveillance bulletins • data: Date of the reported data • code_region: 2-digit code identifying the region (ISTAT nomenclature) • name_region: Name of the region • code_province: 3-digit code identifying the province (ISTAT nomenclature) • name_province: Name of the province • abbreviation_province: Province 2-letter code (ISTAT nomenclature) • lat: Latitude coordinates of the province • long: Longitude coordinates of the province • new_cases: Number of new weekly cases reported • total_cases: Total number of cases reported since the beginning of surveillance 4) wn-ita-equids-surveillance-yyyy.csv • url_bolletins: URL to the surveillance bulletins • data: Date of the reported data • code_region: 2-digit code identifying the region (ISTAT nomenclature) • name_region: Name of the region • code_province: 3-digit code identifying the province (ISTAT nomenclature) • name_province: Name of the province • abbreviation_province: Province 2-letter code (ISTAT nomenclature) • lat: Latitude coordinates of the province • long: Longitude coordinates of the province • new_cases: Number of new weekly cases reported • total_cases: Total number of cases reported since the beginning of surveillance • new_deaths: Number of new weekly deaths reported • total_deaths: Total number of deaths reported since the beginning of surveillance • num_equids_outbreak: Total number of equids present in an outbreak 5) wn-ita-target-birds-surveillance-yyyy.csv and wn-ita-wild-birds-surveillance-yyyy.csv • url_bolletins: URL to the surveillance bulletins • data: Date of the reported data • code_region: 2-digit code identifying the region (ISTAT nomenclature) • name_region: Name of the region • code_province: 3-digit code identifying the province (ISTAT nomenclature) • name_province: Name of the province • abbreviation_province: Province 2-letter code (ISTAT nomenclature) • lat: Latitude coordinates of the province • long: Longitude coordinates of the province • species: Species name of reported cases • new_cases: Number of new weekly cases reported • total_cases: Total number of cases reported since the beginning of surveillance 6) dengue-ita-yyyy.csv/chikungunya-ita-yyyy.csv • data: Date of the reported data • new_cases: Number of new weekly cases reported • total_cases: Total number of cases reported since the beginning of surveillance • new_deaths: Number of new weekly deaths reported • total_cases: Total number of deaths reported since the beginning of surveillance • male: Percentage of weekly cases reported in males • female: Percentage of weekly cases reported in females • median_age: Median weekly age of affected people • autochthonous: Number of weekly cases that are locally transmitted • imported: Number of weekly cases that are imported (acquired from outside the local area) 6) dengue-age-yyyy.csv/chikungunya-age-yyyy.csv • data: Date of the reported data • age: Age of affected people • new_cases: Number of new weekly cases reported • total_cases: Total number of cases reported since the beginning of surveillance • male: Percentage of weekly cases reported in males • female: Percentage of weekly cases reported in females • incidence: Weekly incidence rate of reported cases 7) dengue-ita-regions-yyyy.csv/chikungunya-regions-yyyy.csv • data: Date of the reported data • code_region: 2-digit code identifying the region (ISTAT nomenclature) • name_region: Name of the region • lat: Latitude coordinates of the province • long: Longitude coordinates of the province • new_cases: Number of new weekly cases reported • total_cases: Total number of cases reported since the beginning of surveillance

## 4 Discussion

### 4.1 Environmental and socioeconomic factors influencing arboviruses spread

The spread of arboviruses is significantly influenced by various environmental and socioeconomic factors, the understanding of which is critical to developing effective strategies for epidemic control and prevention. Temperature and rainfall are two critical seasonal factors that directly influence the life cycle and population dynamics of vectors such as mosquitoes (Ciota and Keyel, 2019; Pascoe et al., 2022). For example, transmission of Zika, dengue, and yellow fever viruses is significantly higher during hot and humid seasons in tropical regions (Mordecai et al., 2017; Aliaga‐Samanez et al., 2024). Similarly, abundant rainfall creates more breeding sites by filling containers, ditches and other natural watersheds, facilitating mosquito proliferation (Benedum et al., 2018). Migratory birds contribute significantly to the spread of arboviruses, serving as both reservoirs and vectors. These birds can carry viruses over long distances during their migratory journeys, introducing pathogens to new regions where they can infect local mosquito populations. The study by Ferraguti et al. (Ferraguti et al., 2024) showed that bird migration patterns are aligned with the spread of WNV in Europe. The work, conducted in southwestern Spain, revealed that migratory birds are more likely to be exposed to WNV than native and exotic species. As these birds move between breeding and wintering areas, they can bridge geographic gaps and carry virus across international borders, complicating control efforts. International borders can hinder or facilitate the spread of arboviruses, depending on the effectiveness and coordination of health management and control policies. Well-monitored borders with effective vector control measures can limit the spread of viruses. However, in regions where neighboring countries have divergent resources and policies, the virus can easily cross borders, leading to outbreaks (O'Connor et al., 2021). For example, differences in health infrastructure and disease surveillance between countries can result in undetected transmission, especially when people travel across borders for trade, tourism, or work. Population movements, driven by factors such as seasonal labor migration, displacement due to conflict or environmental changes, can also alter the distribution of arboviruses. These movements can introduce viruses into new areas and increase the frequency and intensity of outbreaks. Refugee camps and temporary settlements often lack adequate vector controls and sanitation facilities, making them hotspots for arbovirus transmission (Kolimenakis et al., 2022). In addition, urbanization and deforestation can change the habitats of vectors, putting them in closer contact with human populations and increasing transmission risks (Tazerji et al., 2022).

### 4.2 Integrated approaches for arboviruses control

Arboviruses epidemics require an integrated and multifactorial approach for effective management (Wallau et al., 2023). Strategies adopted focus on several key aspects, including strengthening epidemiological, entomological, and genomic surveillance, vector control, health system preparedness, public awareness, and advancing scientific research. Epidemiological surveillance is critical for the early identification of human cases of infection, enabling timely interventions to contain the spread of disease. In parallel, entomological surveillance monitors the presence and density of vectors, such as mosquitoes, that are responsible for the transmission of these infections. Genomic information is critical for the early detection of viral outbreaks because it helps to assess the epidemic potential of existing and newly introduced viral strains, to identify the spread of new strains in populations lacking immunity, and to detect the emergence of more virulent variants or those resistant to interventions such as vaccines, antiviral drugs, and various vector control strategies. Vector control is achieved through chemical, biological, and environmental methods (Achee et al., 2019; Huang et al., 2017) aimed at reducing mosquito populations and, consequently, the risk of arboviruses transmission. Health system preparedness is another essential pillar and involves the ongoing training of medical personnel, updating diagnosis and treatment protocols, and adjusting hospital resources to address any outbreaks. Education and public awareness play a crucial role in informing the population about preventive measures and disease symptoms, promoting behaviors that reduce the risk of transmission. In Europe, the approach to arboviruses has had to take into account regional specificities, considering both the species of vectors present and the patterns of disease transmission. The European Union, through the European Centre for Disease Prevention and Control (ECDC), plays a key role in coordinating transnational efforts by providing technical guidelines (https://www.ecdc.europa.eu/sites/default/files/media/en/publications/Publications/TER-Mosquito-surveillance-guidelines.pdf), facilitating information exchange between countries (https://www.ecdc.europa.eu/sites/default/files/documents/Organisation-vector-surveillance-control-Europe_0.pdf), and supporting national and regional preparedness (https://www.ecdc.europa.eu/sites/default/files/documents/ECDC-country-preparedness-2013-2017.pdf). Several European countries have implemented tailored strategies to address specific threats from arboviruses. In Italy, the Ministry of Health has developed a National Plan for Prevention, Surveillance and Response to Arboviruses (https://www.salute.gov.it/imgs/C_17_pubblicazioni_2947_allegato.pdf). This plan focuses on viruses such as Zika, dengue, and chikungunya and includes the following aspects: (i) an early warning system that uses epidemiological and climatic data to predict outbreaks and alert health authorities; (ii) guidelines for diagnosis and treatment of patients, developed in collaboration with infectious disease experts; (iii) strategies for reducing mosquito populations, such as pest control and elimination of breeding sites. France has strengthened its mosquito surveillance network through the use of advanced technologies to monitor mosquito presence in real-time (Calba et al., 2015) and specific interventions in high-risk areas to reduce mosquito populations and prevent outbreaks. Spain has highlighted the need to improve diagnostic capacity by creating standardized procedures for dengue case management. Fernández-Martínez et al. (Fernández-Martínez et al., 2023) conducted a study to assess the risk of local transmission of dengue, focusing on the invasive mosquito *A. albopictus*. They developed a risk assessment tool that combines environmental, entomological, epidemiological, demographic and travel data to map mosquito distribution and potential areas of spread. The study introduced the Index of Travelers from Endemic Areas (IDVZE) to assess the impact of travelers from dengue-prone regions. Some countries have also adopted innovative approaches. For example, Bellini et al. (Bellini et al., 2013) presented a comprehensive study on the application of the sterile insect technique (SIT) (Lees et al., 2015) for controlling *A. albopictus* populations in Italy. The research highlights the importance of weather variables, such as temperature and rainfall, in influencing egg sterility and mosquito density. The findings demonstrate that adjusting gamma ray doses during the sterilization process significantly impacts male mortality rates, ultimately enhancing the efficacy of the SIT program. The collaboration with local authorities and the proactive communication with citizens underscores the community’s support for the initiative. Germany has implemented a surveillance system based on citizen volunteers to monitor the presence of invasive mosquitoes (Pernat et al., 2022). The study found that increased media reporting correlated positively with the number of mosquito samples sent, suggesting that effective communication through mass media significantly increases participant activation. The work identified key factors influencing participation, such as project awareness, attention economy and individual characteristics of potential citizen scientists. The results underscored the importance of strategic media communication in promoting public involvement in scientific research, particularly during periods of heightened mosquito-related public health concern. Significant investment has been devoted to genomic research in the United Kingdom to improve understanding of the spread and evolution of vector-borne viruses. The One Health Vector-Borne Diseases Hub (https://vbdhub.org/) has received £1.5 million in funding, which will facilitate the sharing of data and research findings on vector-borne diseases among researchers and policymakers in the UK (https://www.imperial.ac.uk/news/250073/new-gather-data-diseases-spread-mosquitoes/).

### 4.3 Advances in arboviruses research and vaccine development

At the research and development level, a notable advancement is Valneva’s Ixchiq vaccine for chikungunya, approved under the FDA’s accelerated approval pathway (Branda et al., 2023a). This vaccine represents a significant advancement for individuals over the age of 18 who are at risk. However, for WNV, there is currently no approved vaccine for human use (Cendejas and Goodman, 2024). Although numerous vaccine candidates have entered clinical trials, none have progressed beyond phase II of the FDA’s four-phase licensure process. One prominent candidate, developed by the Vaccine Research Center (VRC), expresses pre-membrane (prM) and envelope (E) glycoproteins and has reached phase I clinical trials, showing neutralizing antibodies in over 90% of young and elderly subjects after three doses. Additionally, a recombinant yellow fever vaccine that also expresses prM and E glycoproteins has demonstrated the ability to generate neutralizing antibodies in 90% of subjects after a single dose. Despite these promising results, challenges remain in developing a human vaccine against WNV. The sporadic nature of WNV outbreaks and high regional variability complicates the logistics of conducting large-scale phase III clinical trials, which require thousands of participants. Moreover, since most individuals infected with WNV are asymptomatic, it is challenging to establish a clear correlation between neutralizing antibody levels and protection. For dengue fever, two vaccines have been licensed: Dengvaxia^®^ (CYD-TDV), developed by Sanofi Pasteur, and Qdenga^®^ (TAK-003), developed by Takeda. Another dengue vaccine, developed by the Laboratory of Infectious Diseases at the National Institutes of Allergy and Infectious Diseases (NIAID) in the United States, is in the late stages of clinical development (https://www.who.int/news-room/questions-and-answers/item/dengue-vaccines).

## 5 Conclusion

The presence of competent mosquito vectors such as *A. albopictus* in Italy has raised concerns about the potential for more widespread transmission. Similar to other Southern European countries, Italy faces a growing threat from arboviruses, exacerbated by climate change and global travel. However, the specific patterns of disease spread, and the effectiveness of control measures can vary significantly. For instance, the 2017 outbreak of chikungunya in Italy, driven by a strain with the E1 226A mutation, reflects a unique adaptation that has not been as prominent in other European outbreaks. The systematic collection and classification of data from ISS have allowed us to create a comprehensive and accessible dataset for the major arboviruses present in Italy, i.e., West Nile, dengue and chikungunya, the details of which are available in the Supplementary Material, that will serve as a necessary tool for public health surveillance and research. The data indicate that WNV has established a significant presence in Italy, with a notable increase in cases over the years (Mingione et al., 2023). Both dengue and chikungunya have shown sporadic outbreaks, with data suggesting a correlation with increased travel and climate variability (Branda et al., 2023b). This trend underscores the need for enhanced vector control measures and public health interventions, particularly in regions with high case numbers.

### 5.1 Limitations

Despite the comprehensive nature of our dataset, some limitations need to be recognized. First, surveillance coverage may vary across regions, leading to potential under-reporting of cases, particularly in less accessible or resource-limited areas. In addition, the sporadic nature of outbreaks, particularly for chikungunya, can make it difficult to establish clear transmission patterns and predict future outbreaks. Another limitation is the reliance on existing public health infrastructure, which may not be fully equipped to deal with rapid changes in disease patterns due to climate change. In addition, the genetic variability of arboviruses and their vectors presents a challenge for developing targeted interventions that remain effective over time.

### 5.2 Future perspectives

Future research should focus on improving understanding of how climate change affects arbovirus transmission and vector behavior. Detailed genomic analyses are critical to identify mutations that impact virus transmission, virulence, and resistance to control methods. In addition, advanced climate modeling is needed to predict changes in vector habitats, enabling more proactive interventions. There is also a need to develop integrated surveillance platforms that combine health, environmental, and genomic data. These platforms can improve real-time monitoring and response capabilities, especially in regions with rapidly changing vector dynamics. Given the continuing threat of arboviruses, future strategies should prioritize strengthening early detection systems that integrate epidemiological, environmental, and genomic data for faster response to outbreaks. This requires not only national efforts but also greater international cooperation, as sharing data and best practices can significantly improve global preparedness. Another important area for future research is the development of new vector control technologies and sustainable methods, such as genetic approaches to reduce mosquito populations or the development of more efficient biocontrol methods.

Climate change poses a growing challenge in the fight against arboviruses as warming temperatures and altered rainfall patterns create environments more conducive to mosquito proliferation. To address these climate-related problems, health policies must be adapted to anticipate long-term changes in disease transmission patterns. Governments should invest in building climate-resilient health systems that can withstand more frequent and intense arbovirus outbreaks. This includes increasing funding for public health infrastructure, training health workers, and improving vector control measures, particularly in high-risk areas. Mitigation efforts should also focus on public education, encouraging communities to reduce mosquito breeding sites and adopt personal protective measures to avoid mosquito bites. The use of advanced technologies, such as climate monitoring systems and predictive modeling tools, can help predict outbreaks and plan early interventions. Finally, continued international collaboration will be essential to manage the cross-border spread of arboviruses, especially as climate-driven migration increases the risk of introducing new diseases to previously unaffected regions.

## Data Availability

The datasets presented in this study can be found in online repositories. The names of the repository/repositories and accession number(s) can be found in the article/Supplementary Material.
