# Corrigendum: ArboItaly: leveraging open data for enhanced arbovirus surveillance in Italy

**DOI:** 10.3389/fphar.2024.1517547

**Published:** 2024-11-06

**Authors:** Francesco Branda, Marta Giovanetti, Giancarlo Ceccarelli, Massimo Ciccozzi, Fabio Scarpa

**Affiliations:** ^1^ Unit of Medical Statistics and Molecular Epidemiology, Università Campus Bio-Medico di Roma, Rome, Italy; ^2^ Department of Sciences and Technologies for Sustainable Development and One Health, Università Campus Bio-Medico di Roma, Rome, Italy; ^3^ Instituto Rene Rachou, Fundação Oswaldo Cruz, Belo Horizonte, Brazil; ^4^ Climate Amplified Diseases and Epidemics (CLIMADE), Brasilia, Brazil; ^5^ Department of Public Health and Infectious Diseases, University Hospital Policlinico Umberto I, Sapienza University of Rome, Rome, Italy; ^6^ Department of Biomedical Sciences, University of Sassari, Sassari, Italy

**Keywords:** arboviruses, Italy, open data, west nile, chikungunya, dengue, public health, surveillance

In the published article, there was an error in the **Funding** statement. The statement erroneously declared that no financial support was received for the research, authorship, and/or publication of this article. The correct Funding statement appears below.

