# Supplementary material for: ArboItaly: Leveraging open data for enhanced arbovirus surveillance in Italy
Source: Front Pharmacol. 2024 Sep 23;15:1459408. doi: 10.3389/fphar.2024.1459408 (PMC11456481; doi:10.3389/fphar.2024.1459408)
Supplement: Supplementary file 1 [file DataSheet1.docx]

Supplementary Material

ArboItaly: Leveraging open data for enhanced arbovirus surveillance in Italy

Francesco Branda^1*^, Marta Giovanetti^2,3,4^, Giancarlo Ceccarelli^5^, Massimo Ciccozzi^1^, Fabio Scarpa^6^

1. Unit of Medical Statistics and Molecular Epidemiology, Università Campus Bio-Medico di Roma, Rome, Italy
2. Department of Sciences and Technologies for Sustainable Development and One Health, Università Campus Bio-Medico di Roma, Italy
3. Instituto Rene Rachou, Fundação Oswaldo Cruz, Minas Gerais, Brazil;
4. Climate Amplified Diseases and Epidemics (CLIMADE), Brazil, Americas
5. Department of Public Health and Infectious Diseases, University Hospital Policlinico Umberto I, Sapienza University of Rome, Rome, Italy
6. Department of Biomedical Sciences, University of Sassari, Sassari, Italy

*** Correspondence:** f.branda@unicampus.it

# Chikungunya

# Chikungunya virus (CHIKV), an alphavirus transmitted by mosquitoes of the *Aedes* species, was originally identified in Tanzania in 1952 and belongs to the *Togaviridae* family, genus *Alphavirus*. Due to the severe arthritic symptoms, the disease was named "chikungunya," meaning "bent over" in the Makonde language. Following this initial outbreak, several epidemics were described between in various African, and Asian countries. In Europe, the first autochthonous CHIKV outbreak was reported in Italy in 2007. Additionally, a significant number of cases in France were confirmed in the years 2010-2014. In the Americas, the emergence of CHIKV occurred in 2013 on the island of Saint Martin in the Caribbean, and since then, several new cases have been identified in 45 countries in the Caribbean, South America, and Central America [1]. Phylogenetic analyses have revealed that CHIKV presents four genotypes (or lineages), named according to their geographic distribution, namely the West African genotype, the Asian genotype, the East-Central-South African (ECSA) genotype, and the Indian Ocean (IOL) genotype. Studies have shown that various strains of the IOL CHIKV have undergone a series of mutations, including the adaptive mutation E1-A226V, which confers an increased replication rate in Aedes albopictus, thus enhancing the potential for viral transmission [1]. Clinical manifestations of CHIKV infection include non-specific flu-like symptoms, distinctive rash, and prolonged joint discomfort, persisting beyond the acute phase of the illness. Elevated mortality rates have been documented during large-scale CHIKV outbreaks [1]. Dysregulation of innate defense mechanisms, notably the cytokine inflammatory response, may contribute to the pathogenesis of clinical symptoms and the establishment of chronic disease. While no specific treatment exists for CHIKV infection, prevention through vector control and avoiding mosquito bites remains paramount. Diagnosis typically involves molecular assays or virus culture in the early stages, with serological testing utilized in later stages. Differential diagnosis should consider potential cross-reactivity with other viruses within the same antigenic complex, such as O'nyong-nyong virus. As of August 2017, locally acquired cases of CHIKV were reported in Southeast France and several regions of Italy [2]. Rigorous vector control measures, coupled with environmental factors such as declining temperatures, facilitated the containment of these outbreaks. In Italy, the 2017 outbreak was attributed to CHIKV expressing the E1 226A mutation, capable of sustained local transmission in areas where *Ae*. *albopictus* is the primary vector. From January 1st to June 10th, 2024, the national arbovirus surveillance system reported four confirmed cases of chikungunya, all associated with travel abroad, and no fatalities (https://www.epicentro.iss.it/chikungunya/aggiornamenti). Active monitoring is pivotal to face the emerging threat posed by climate change and the likely reemergence of mosquito-borne pathogens in Italy and across the globe.

# Dengue

# The dengue virus (DENV) has spread widely over the past decade and now poses a threat to about one-third of the global human population, mainly affecting inhabitants of tropical and subtropical regions where mosquito vectors of the *Aedes* genus are widely distributed [3]. Increased human mobility, population growth, unplanned urbanization, globalization, climate change, and unsuccessful vector control programs have all contributed to the expansion of DENV, making it a major global public health threat [4]. DENV is a single-stranded positive-sense RNA virus with a genome of approximately 11,000 kb belonging to the *Flaviviridae* family (genus *Orthoflavivirus*) [4]. Its genome encodes a polyprotein that is post-translationally processed into three structural proteins (capsid, pre-membrane or membrane, and envelope) and seven non-structural proteins (NS1, NS2a, NS2b, NS3, NS4a, NS4b, and NS5). DENV is classified into four antigenically distinct and genetically related serotypes (DENV1-4). So far, 19 genotypes of DENV have been identified, with five in DENV1 (1I-V), six in DENV2 (2I-VI), and four each in DENV3 (3I, 3II, 3III, 3V) and DENV4 (4I-IV) [5]. Since heterotypic infections are the major risk factor for severe clinical outcomes, surveillance of circulating serotype diversity is crucial for public health. Dengue continues to pose a significant challenge to public health in Brazil, as evidenced by a recent increase in cases, reaching 6.148 million reported cases and resulting in 4,207 deaths as of June 27, 2024 (https://www.gov.br/saude/pt-br/assuntos/saude-de-a-a-z/a/aedes-aegypti/monitoramento-das-arboviroses). During this period, the country observed the co-circulation of different serotypes (DENV1, DENV2, and the reintroduction of DENV3 cases – detected in both the northern and southern regions of Brazil, with the latter being an imported case from Suriname), highlighting the importance of active surveillance. Furthermore, this situation underscores the role of climate change and human mobility in disease transmission. Globally, dengue virus circulation is increasing, with heightened epidemic activity in previously unaffected countries, including within the European region [6]. In 2023, global dengue activity peaked, and Italy reported the highest number of dengue cases and local chains of transmission to date. By integrating geographic, epidemiological, genomic, and climatic spatio-temporal data, a recent study provided an overview regarding the dengue viral transmission patterns over the past eight years in Italy. This includes analysis of circulating viral lineages, geographic distribution, reporting hotspots, and the potential influence of local climate.

# West Nile

# The West Nile virus (WNV), a member of the Flaviviridae family, was initially identified in the West Nile district of Uganda in 1937 [7]. Currently, WNV is widely found in various regions, including Africa, Europe, North America, the Middle East, and Asia [8]. Its transmission mainly occurs through a cycle between mosquitoes and birds, with mosquitoes of the Culex genus, especially Cx. pipiens and Cx. quinquefasciatus, considered the primary vectors [9]. Although WNV can infect humans, horses, and other mammals, the latter are considered "dead-end" hosts due to their low capacity to spread the infection further. In most cases, about 80% of human infections are asymptomatic, while the remainder may develop mild or severe symptoms [9]. Mild symptoms include fever, headache, fatigue, and vomiting, while severe cases, known as neuroinvasive disease, are characterized by high fever, coma, seizures, and paralysis. Although less common, infections in horses can lead to neurological diseases and even death, making them important sentinels. Several factors may contribute to the silent circulation of WNV in the country, including the lack of active surveillance, rates of mild human disease, co-circulation of other mosquito-transmitted viruses, and late diagnoses in animals and humans, hindering viral detection [10]. WNV diversity has been investigated through sequencing and phylogenetic analysis of complete genomes, resulting in the identification of 9 lineages to date. Among them, WNV lineage 1 (WNV L1) and WNV lineage 2 (WNV L2) are by far the most widely distributed and virulent, capable of causing numerous cases worldwide [11]. The West Nile virus (WNV) has spread throughout continental Europe, most notably in Northern Italy's Po River Valley [12]. During the summer of 2022, Italy saw an extraordinary increase in the number of cases of WNV infections, including the primary sequelae (West Nile fever (WNF) and West Nile neuroinvasive disease (WNND). Recent studies have pointed out that atypical weather was associated with the 2022 early start of West Nile virus transmission in Italy, reinforcing the importance of an integrated system to face the challenges due to climatic changes [10].

# Usage Notes

To illustrate the value and potential of this data, we present three examples of key analyses.

**Example 1. Temporal and geospatial analysis of arboviruses incidence**

Understanding the seasonal dynamics, particularly the peaks during warmer months when mosquito activity is heightened, is crucial for timing effective public health interventions, including targeted mosquito control measures and awareness campaigns. Researchers can analyze month-by-month or quarter-by-quarter data to unveil seasonal patterns of disease incidence, especially for mosquito-borne viruses like West Nile. This understanding enables timely interventions, such as mosquito control and public health messaging during transmission peaks. Additionally, examining year-to-year variations and employing statistical models can identify trends in disease incidence, elucidating factors behind outbreaks such as climate change, urbanization, and global travel patterns. Identifying regions with persistently high incidence rates, such as Lombardy for both Dengue and West Nile, allows for focused resource allocation and tailored interventions, thereby enhancing effectiveness in disease control and reducing healthcare system burdens. Comparative analysis between different arboviruses within the same region and across different regions offers valuable insights into local transmission dynamics and the efficacy of control measures, refining strategies to mitigate risks and safeguard public health.
Figure 1 shows the annual incidence rates of the three arboviruses analyzed in this study from 2015 to 2023. In particular, the incidence of West Nile in Italy (Figure1B1) exhibits significant variability across the years, with a notable peak observed in 2018 (1,491 cases). Subsequent years show fluctuations, indicating varying levels of transmission intensity. Regions such as Lombardy and Veneto consistently report higher case numbers compared to other regions, suggesting these areas may serve as potential hotspots for the virus. Dengue cases in Italy (Figure1B2) are relatively lower compared to West Nile virus, but there are noticeable peaks in regions like Lombardy and Lazio. The highest incidence occurred in 2023 (377 cases), indicating a need for enhanced monitoring and possibly increased vector control measures in affected regions. The fluctuating pattern of Dengue cases suggests potential factors such as travel-related cases, changes in climate influencing mosquito distribution, or other environmental factors that may contribute to sporadic outbreaks. Chikungunya cases in Italy (Figure1B3) show a fluctuating pattern with occasional peaks observed in regions like Emilia-Romagna and Lombardy. This sporadic incidence suggests outbreaks rather than sustained transmission, highlighting the importance of maintaining vigilant surveillance and rapid response capabilities.

**A.**


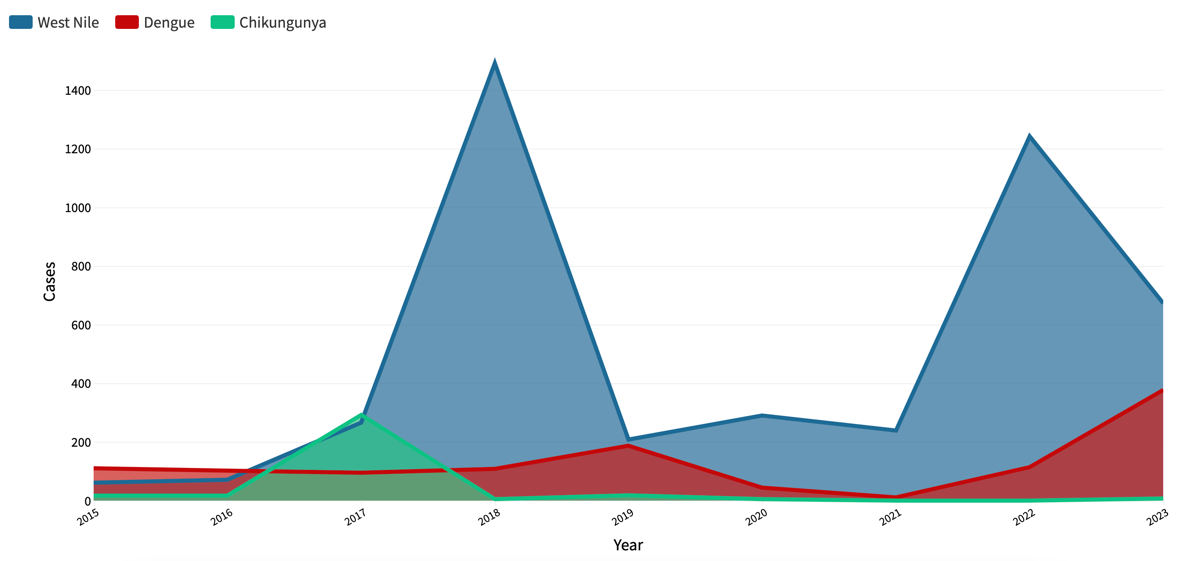


**B1.** **B2. B3.**


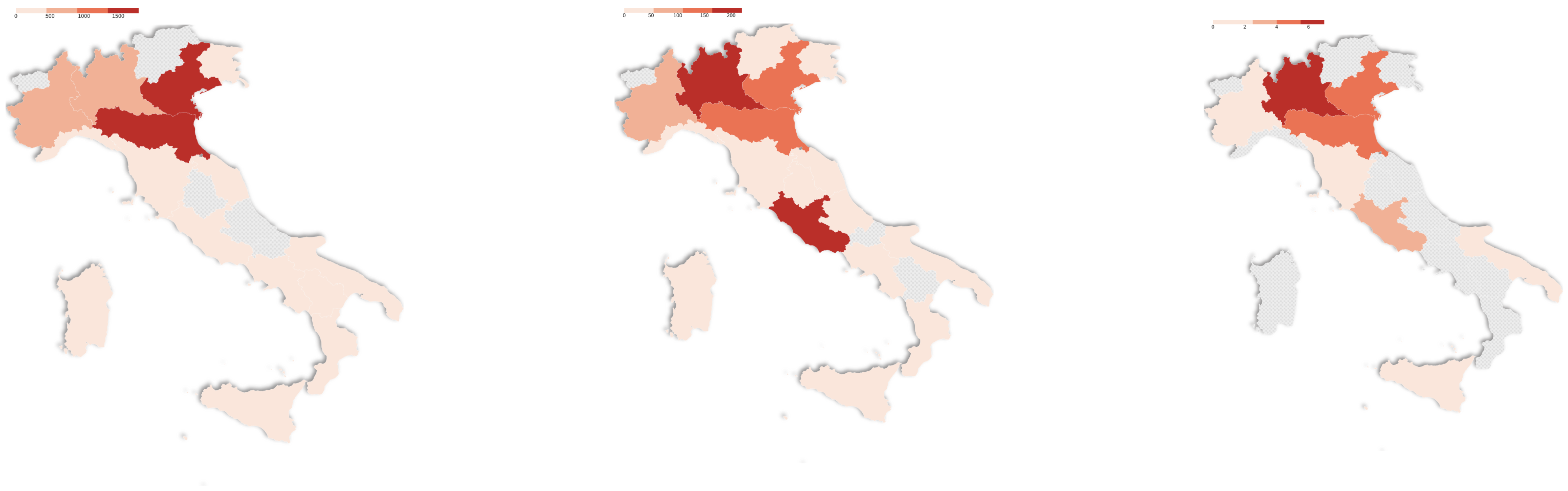

**Figure 1. The distribution of outbreaks through time. A) Number of cases per year by arbovirus. B1) Distribution of West Nile cases per region (2015 – 2023); B2) Distribution of Dengue cases per region (2018 – 2023); B3) Distribution of Chikungunya cases per region (2018 – 2023).**

**Example 2. Exploring factors influencing the spread of arboviruses**

Understanding the complex dynamics of arbovirus spread requires advanced data analytics techniques that integrate diverse datasets and factors. By analyzing climate variables, vector populations, and human demographics, researchers can uncover intricate relationships and identify key factors that influence transmission patterns. For example, our analysis reveals several insights into the factors influencing the incidence of new weekly West Nile cases. Considering climatic variables and geographic coordinates, a strong effect of geographic location emerges. Figure 2 describes how geographic coordinates provide a powerful tool for discerning geographic patterns and spatial clusters of arbovirus transmission. Specifically, latitude shows a significant negative association, with a decrease of approximately 0.22579 new cases per unit increase (t-value = -4.223, p < 0.001). In contrast, longitude shows a significant positive association, with an increase of about 0.18619 new cases per unit (t-value = 3.555, p = 0.000436). These results point to marked geographic patterns in arbovirus transmission, indicating regions where incidence rates vary greatly by coordinates. Climatic variables show fewer clear associations with new weekly cases. Temperature, humidity and precipitation do not reach statistical significance (p = 0.645, p = 0.298,
p = 0.555 respectively). These results suggest that, in the specific context analyzed, the direct influence of climatic factors on arbovirus transmission may be more complex than initially assumed. The apparent lack of significant correlations does not necessarily imply the irrelevance of these variables, but rather indicates the need for further analysis. It may be necessary to consider nonlinear interactions, threshold effects or temporal variations to fully capture the role of climate in arbovirus transmission dynamics. In addition, these results underscore the importance of integrating other environmental and socio-ecological factors to gain a more complete understanding of the mechanisms of disease spread.
In summary, the modest adjusted R-squared value of 0.156 indicates that the model explains 15.6% of the variance in new cases, suggesting that other factors beyond those included in the model are influencing transmission dynamics. This underscores the complexity of arbovirus epidemiology and the need for comprehensive, context-specific analyses that integrate additional variables and account for local conditions.


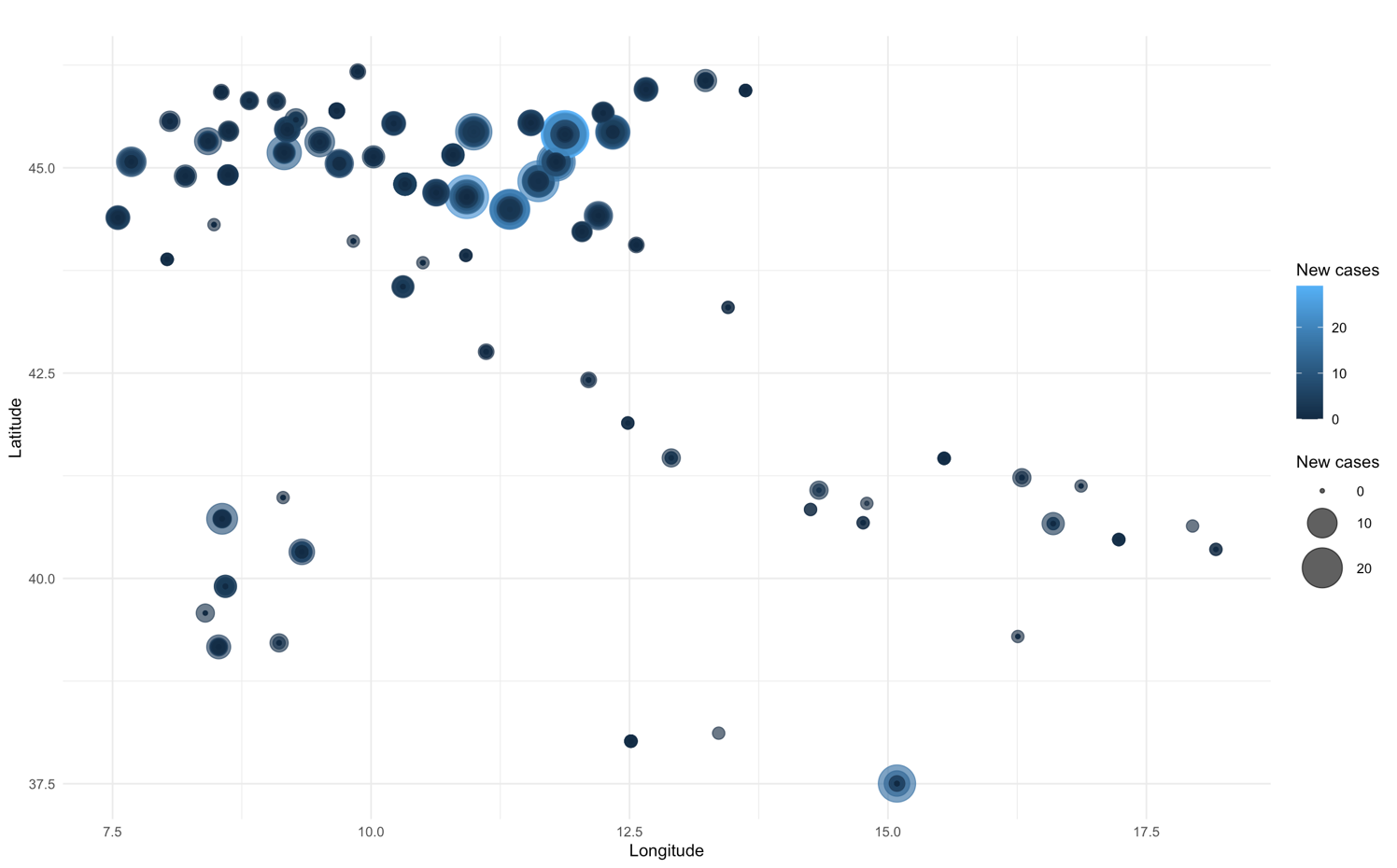


**Figure 2. Geographic distribution of new West Nile cases based on latitude and longitude variables.**

**Example 3. Predicting the future spread of arboviruses**

Modeling techniques to predict arbovirus spread include a wide range of approaches, from traditional mathematical models to the latest innovations in machine learning. Mathematical and statistical models provide the foundation, using differential equations and statistical analysis to describe transmission dynamics. Spatiotemporal models integrate geographic and temporal data, enabling more accurate predictions on a regional scale. The advent of machine learning has revolutionized the field, enabling analysis of complex data sets and identification of nonlinear patterns. Agent-based models offer a microscopic approach, simulating interactions between individual vectors, pathogens, and hosts. Big data analysis, combining sources as diverse as satellite data and social media, is becoming increasingly crucial. These techniques, often integrated into hybrid approaches, are constantly improving our ability to predict and respond to arbovirus threats. For example, Figure 3 shows the application of ARIMA (AutoRegressive Integrated Moving Average) model, a time series analysis technique that combines autoregressive (AR) components, which use past values to predict future ones, with moving average (MA) components, which use past forecast errors in a regression-like model. The ARIMA model was applied to the two provinces that recorded the highest number of West Nile virus cases in Italy (i.e., Venezia and Bologna). In particular, Figure 3A1 shows the time series of West Nile virus cases over a period of 500 time units in Venice. The time series shows a clear cyclic pattern with regular peaks reaching about 40-50 cases. These peaks are interspersed with periods of low numbers of cases, suggesting a strong seasonal component in disease transmission. The ARIMA model seems to capture this seasonality well, as shown by the regular and uniform fluctuations in the predicted values. Figure3A2 shows the time series over 400 time units in Bologna, with the number of cases ranging from 0 to 60. Unlike Venice, Bologna's time series shows a more erratic behavior, with sharp and irregular peaks in the number of cases. The peaks in Bologna reach higher values, up to about 60 cases, but occur in a less predictable manner. The application of the ARIMA model to these two distinct time series demonstrates its flexibility in capturing different patterns of disease transmission. For Venice, the model probably emphasizes the seasonal component (AR), while for Bologna it may rely more on the moving average (MA) component to explain sudden spikes and irregularities. The contrasting patterns between the two provinces underscore the importance of local factors in arbovirus transmission and the need for predictive models tailored to different geographic areas.

**A1.**

**A2.**


**Figure 3. Example of using the ARIMA model on Italian provinces. The black line represents the actual cases, while the blue line indicates the model predictions. A1) Venezia; A2) Bologna.**

# References

Xavier J, Alcantara LC, Fonseca V, Lima M, Castro E, Fritsch H, Oliveira C, Guimarães N, Adelino T, Evaristo M, Rodrigues ES. Increased interregional virus exchange and nucleotide diversity outline the expansion of chikungunya virus in Brazil. Nature Communications. 2023 Jul 21;14(1):4413.

Lindh E, Argentini C, Remoli ME, Fortuna C, Faggioni G, Benedetti E, Amendola A, Marsili G, Lista F, Rezza G, Venturi G. The Italian 2017 outbreak chikungunya virus belongs to an emerging Aedes albopictus–adapted virus cluster introduced from the Indian subcontinent. InOpen forum infectious diseases 2019 Jan (Vol. 6, No. 1, p. ofy321). US: Oxford University Press.

Adelino TÉ, Giovanetti M, Fonseca V, Xavier J, de Abreu ÁS, do Nascimento VA, Demarchi LH, Oliveira MA, da Silva VL, de Mello AL, Cunha GM. Field and classroom initiatives for portable sequence-based monitoring of dengue virus in Brazil. Nature communications. 2021 Apr 16;12(1):2296.

Giovanetti M, Pereira LA, Santiago GA, Fonseca V, Mendoza MP, de Oliveira C, de Moraes L, Xavier J, Tosta S, Fristch H, de Castro Barbosa E. Emergence of dengue virus serotype 2 cosmopolitan genotype, Brazil. Emerging infectious diseases. 2022 Aug;28(8):1725.

Waman VP, Kolekar P, Ramtirthkar MR, Kale MM, Kulkarni-Kale U. Analysis of genotype diversity and evolution of Dengue virus serotype 2 using complete genomes. PeerJ. 2016 Aug 24;4:e2326.

Zatta M, Brichler S, Vindrios W, Melica G, Gallien S. Autochthonous dengue outbreak, paris region, France, september–october 2023. Emerging Infectious Diseases. 2023 Dec;29(12):2538.

Fritsch H, Pereira FM, Costa EA, Fonseca V, Tosta S, Xavier J, Levy F, Oliveira CD, Menezes G, Lima J, Santos L. Retrospective investigation in horses with encephalitis reveals unnoticed circulation of West Nile virus in Brazil. Viruses. 2022 Jul 14;14(7):1540.

Mingione M, Branda F, Maruotti A, Ciccozzi M, Mazzoli S. Monitoring the West Nile virus outbreaks in Italy using open access data. Scientific Data. 2023 Nov 7;10(1):777.

Costa ÉA, Giovanetti M, Silva Catenacci L, Fonseca V, Aburjaile FF, Chalhoub FL, Xavier J, Campos de Melo Iani F, da Cunha e Silva Vieira MA, Freitas Henriques D, Medeiros DB. West nile virus in brazil. Pathogens. 2021 Jul 15;10(7):896.

Lourenço J, Pinotti F, Nakase T, Giovanetti M, Obolski U. Atypical weather is associated with the 2022 early start of West Nile virus transmission in Italy. Eurosurveillance. 2022 Aug 25;27(34):2200662.

Bakonyi T, Ivanics É, Erdélyi K, Ursu K, Ferenczi E, Weissenböck H, Nowotny N. Lineage 1 and 2 strains of encephalitic West Nile virus, central Europe. Emerging infectious diseases. 2006 Apr;12(4):618.

Riccò M, Zaniboni A, Satta E, Ranzieri S, Cerviere MP, Marchesi F, Peruzzi S. West Nile virus infection: a cross-sectional study on Italian medical professionals during summer season 2022. Tropical Medicine and Infectious Disease. 2022 Nov 28;7(12):404.
